# In-hospital mortality of patients admitted to the intermediate care unit in hospitals with and without an intensive care unit: a nationwide inpatient database study

**DOI:** 10.1186/s13054-025-05275-0

**Published:** 2025-01-20

**Authors:** Hiroyuki Ohbe, Daisuke Kudo, Yuya Kimura, Hiroki Matsui, Hideo Yasunaga, Shigeki Kushimoto

**Affiliations:** 1 Department of Emergency and Critical Care Medicine, Tohoku University Hospital, 1-1 Seiryo-Machi, Aoba-Ku, Sendai, 980-8574 Japan; 2 Department of Clinical Epidemiology and Health Economics, School of Public Health, The University of Tokyo, 7-3-1 Hongo, Bunkyo-Ku, Tokyo, 113-0033 Japan; 3 Division of Emergency and Critical Care Medicine, Tohoku University Graduate School of Medicine, 2-1 Seiryo-Machi, Aoba-Ku, Sendai, Miyagi 980-8575 Japan; 4 Department of Health Services Research, Graduate School of Medicine, The University of Tokyo, 7-3-1 Hongo, Bunkyo-Ku, Tokyo, 113-0033 Japan

**Keywords:** Intermediate care unit, Intensive care unit, Critical care utilization, Stepdown unit, Japan

## Abstract

**Background:**

Intermediate care units (IMCUs) provide care for patients who need more intensive treatment than general wards but less than intensive care units (ICUs). Although the concept of an IMCU requires co-location with an ICU, some hospitals have IMCUs but no ICUs, which potentially worsens patient outcomes. This study aimed to examine the annual trends and care processes, and compare the outcomes of patients admitted to IMCUs in hospitals with and without ICUs using a nationwide inpatient database in Japan.

**Methods:**

This retrospective cohort study was conducted from 2016 to 2022 using the Diagnosis Procedure Combination Database and Hospital Bed Function Reports in Japan. The main exposure was admission to the IMCU in hospitals with and without ICUs. The primary outcome was in-hospital mortality rate in hospitals with and without ICUs that were compared using multilevel logistic regression models adjusted for confounders.

**Results:**

The number of IMCU beds in hospitals without ICUs increased by 59% from 3,388 in 2016 to 5,403 in 2022, and the IMCU beds in hospitals without ICUs represented 38% (n = 5,403/14,185) of all IMCU beds in Japan in 2022. Among the 3,061,960 eligible patients in the IMCUs, 2,296,939 (75%) and 765,021 (25%) were admitted to hospitals with and without ICUs, respectively. Transfer between IMCUs and ICUs occurred for 10.5% (n = 320,938/3,061,960) of patients, with a large variability between hospitals. After adjusting for potential confounders, patients admitted to IMCUs in hospitals without ICUs had significantly higher in-hospital mortality than those in hospitals with ICUs (adjusted odds ratio: 1.15; 95% confidence interval: 1.10–1.20; *p* < 0.001).

**Conclusions:**

Admission in IMCUs in hospitals without ICUs increased, but was associated with higher in-hospital mortality. These findings suggest that IMCUs should be placed in hospitals with ICUs.

**Supplementary Information:**

The online version contains supplementary material available at 10.1186/s13054-025-05275-0.

## Background

Intensive care units (ICUs) provide life-saving treatment for critically ill patients and have become essential components of modern hospital care. However, ICUs have highly limited medical resources and are costly; therefore, the utilization of ICU resources must be both equitable and optimized. Guidelines for ICU admission and discharge have been developed to improve the efficiency of ICU resource utilization in countries, such as the United States [1], United Kingdom [2], India [3], and Japan [4].

Intermediate care units (IMCUs), also referred to as high-dependency care units or step-down units, provide care for patients who do not require the intensive human and medical resources of an ICU, but still need care beyond that provided by general wards [5]. Because many patients in ICUs do not require active interventions [6, 7], IMCUs were initially designed to safely manage such patients and to improve access to ICUs for more critically ill patients. Therefore, the key concepts of IMCUs revolve around enhancing access to ICUs for patients with more severe conditions without worsening the health outcomes of patients transferred and admitted to IMCUs [5]. These concepts require co-location with the ICU in the same hospital, and IMCUs are provided in a dedicated stand-alone unit or embedded within ICUs or general wards in Europe and the United States [5, 8]. The ICU admission and discharge guidelines in India recommend the establishment of IMCUs within hospitals with ICUs [3].

IMCUs have been increasingly used in recent years because hospitals have established more IMCUs to manage complex patients, optimize resources, and reduce costs [8, 9]. However, in some countries, including Japan, some hospitals without ICUs have IMCUs [10], which raises the question of whether the structure of IMCUs in hospitals without ICUs worsens health outcomes for critically ill patients. To the best of our knowledge, no study has examined this clinical question, which can provide important insights for physicians and policymakers to improve the critical care system.

This study aimed to describe the temporal changes in the number of IMCU and ICU beds in Japan from 2016 to 2022 and compare the outcomes of patients admitted to IMCUs in hospitals with and without ICUs, using a nationwide inpatient database in Japan.

## Methods

### Data source

This nationwide retrospective cohort study utilized data from the Diagnosis Procedure Combination (DPC) database and Hospital Bed Function Report 2016–2022 in Japan. The DPC database includes discharge abstracts and administrative claims data from more than 1,500 voluntarily participating acute-care hospitals in Japan and accounts for nearly 70% of the acute-care hospital beds across the country [11]. The database contains detailed patient-level data for all hospitalizations, such as age, sex, route of admission, diagnoses recorded using the International Classification of Diseases, Tenth Revision codes, daily procedures, daily drug administrations, and discharge status. In a previous study that examined the validity of recorded procedures and diagnoses, both the sensitivity and specificity of the procedures exceeded 90%, whereas the sensitivity and specificity of the primary diagnoses were 78.9% and 93.2%, respectively [12].

We also used the Hospital Bed Function Report 2016–2022, which is published annually by the Ministry of Health, Labour, and Welfare in Japan and includes functional information and statistics of facilities as of July 1 in each year [10]. Publicly available data included the types of hospitals (e.g., academic hospitals or tertiary emergency hospitals), types of wards (e.g., general ward, ICU, or IMCU), and number of licensed hospital beds in each ward. We combined this information with the data from the DPC database using a specific hospital identifier and fiscal year.

The Institutional Review Board of The University of Tokyo approved this study (approval number: 3501–5; approval date: May 19, 2021). Because all data were de-identified, the requirement for informed consent was waived. The study procedures were conducted in accordance with the tenets of the Declaration of Helsinki.

### Definition of ICU and IMCU

Under the national health insurance in Japan, ICU was defined as a separate unit providing critical care services with at least one physician on site 24 h per day, at least two intensivists (procedure codes A3011 and A3012 only), around-the-clock nursing with a nurse-to-patient ratio of > 1:2, ICU nurse (A3011 and A3012 only), a clinical engineer in the hospital 24 h per day (A3011 and A3012 only), and equipment necessary to care for critically ill patients [13]. IMCU was defined similarly to ICU but does not require an intensivist staff and ICU nurse, and the nurse-to-patient ratio required is 1:3, 1:4, or 1:5 [5]. Additional details and the Japanese medical procedure codes used to define ICU and IMCU are listed in Supplementary Table 1, Additional File 1. We did not include patients admitted to the neonatal or obstetric ICU under the definition of ICU or IMCU.

### Study population

In the analysis of the DPC database, we included patients who were admitted to IMCUs between April 1, 2016 and March 31, 2023. We excluded patients from hospitals that could not be combined with data from the Hospital Bed Function Report 2016 and 2022, and patients from hospitals that did not have any IMCU beds according to the Hospital Bed Function Report. Data from all hospitals and their wards were used to analyze the Hospital Bed Function Report.

### Exposure, process, and outcome measurements

The main exposure variable was admission to the IMCU in hospitals with and without ICUs.

The process measurements included transfer between ICU and IMCU, step-down transfer from ICU to IMCU, step-up transfer from IMCU to ICU, hospital transfer directly from IMCU, and life-sustaining therapies during IMCU stay. Life-sustaining therapies included invasive mechanical ventilation, vasopressors (noradrenaline or adrenaline), cardiopulmonary resuscitation, mechanical circularity support, and renal replacement therapies. ICU admission guidelines recommend that these patients receiving life-sustaining therapies be admitted to the ICU and not the IMCU [1–4].

The primary outcome was in-hospital mortality rate. Secondary outcomes included IMCU mortality, discharge destination, length of IMCU stay, length of hospital stay, and total hospitalization cost. The DPC database included estimated costs based on reference prices from the Japanese national fee schedule, which determines item-by-item prices for consultations, oral drugs, injections, procedures, surgery and/or anesthesia, tests, radiology, hospital fees, diet, and other inpatient services [11]. In this study, the total hospitalization cost was calculated based on the aggregation of all estimated costs reimbursed for inpatient care at the hospital.

### Statistical analysis

Continuous variables are summarized as means with standard deviation (SD) or medians with interquartile ranges, as appropriate. Categorical variables are expressed as frequencies and percentages. Due to the large sample size in this study, comparisons between IMCUs in hospitals with and without ICUs were performed using standardized mean differences (SMD), with an absolute SMD of ≤ 10% denoting a negligible imbalance between the two groups [14].

To examine trends from 2016 to 2022, we performed the Jonckheere–Terpstra trend test for continuous variables and the Cochran–Armitage trend test for binomial proportions across fiscal years [15].

We used multilevel mixed-effects logistic regression models with random intercepts to compare in-hospital mortality between patients admitted to the IMCU in hospitals with and without ICUs [16]. The analysis accounted for the hierarchical structure of the data with patients nested within hospitals. Patient-level covariates included fiscal year, age, sex, Charlson Comorbidity Index, Japan Coma Scale score at admission, location before hospitalization, admission classification, length of hospital stay before IMCU admission, main etiology of admission, and procedures on the day of IMCU admission that were used in the validated procedure-based organ failure assessment model for risk adjustment in the DPC database [17]. Odds ratios with 95% confidence intervals (CI) were reported. We also used multilevel mixed-effects regression models to analyze hospitalization costs. As hospitalization cost was a skewed outcome, a log-transformed cost was applied to the model, which yielded relative risk.

Two subgroup analyses were performed: one based on patient age (≥ 75 or < 75 years) [18] and the second based on whether patients required life-sustaining therapies or not during IMCU stay. To assess heterogeneity in the effect of IMCU admission in hospitals without ICU on outcomes between the subgroups, we included interaction terms between exposure and these variables in the model and evaluated statistical significance using p for interaction.

Three sensitivity analyses were performed. First, we categorized patients in hospitals with both IMCU and ICU into quartiles according to the proportion of hospital transfer between the IMCU and ICU. Second, to account for potential biases arising from differences in patient case-mix, we performed a propensity score matching analysis. We first computed the propensity scores for patients admitted to the IMCU in hospitals without ICU using a multivariable logistic regression model that included all the aforementioned covariates. Then, we performed a one-to-one nearest-neighbor matching without replacement for the estimated propensity scores, using a caliper width set at 20% of the standard deviation of the propensity scores. After the propensity score matching, we used multilevel mixed-effects regression models to compare outcomes. Third, to account for potential biases arising from treatment limitations, we excluded patients who died without receiving invasive mechanical ventilation.

Statistical analyses were performed using a two-sided p-value at a 5% significance level. All analyses were performed using Stata/MP, Version 18.0 (StataCorp, College Station, TX, USA).

## Results

Analysis of the Hospital Bed Function Report from 2016 to 2022 showed that the number of IMCU beds in Japan significantly increased by 32% (p for trend = 0.004) from 10,736 beds (703 hospitals) in 2016 to 14,185 beds (856 hospitals) in 2022 (Table 1). The number of IMCU beds in hospitals without ICUs exhibited a 59% increase from 3,388 to 5,403 beds. In 2022, IMCU beds in hospitals without ICUs accounted for 38% (5,403/14,185) of all IMCU beds in Japan. In contrast, the number of ICU beds significantly decreased by 7% (p for trend = 0.024), from 7334 beds (655 hospitals) in 2016 to 6805 beds (577 hospitals) in 2022. The ICU beds in hospitals with both ICUs and IMCUs decreased by 36% (from 2,373 to 1,523 beds).

**Table 1:** Analysis of the Hospital Bed Function Reports from 2016 to 2022

	Fiscal year							Change from 2016 to 2022	*p* for trend*
	2016	2017	2018	2019	2020	2021	2022		
Number of unit beds									
Total number of IMCU beds	10,736	11,418	11,934	12,627	13,003	14,791	14,185	32%	0.004
IMCU beds in hospitals with IMCU but without ICU	3,388	3,779	4,008	4,421	4,528	5,262	5,403	59%	0.002
IMCU beds in hospitals with both IMCU and ICU	7,348	7,639	7,926	8,206	8,475	9,529	8,782	20%	0.004
Total number of ICU beds	7,334	7,109	7,059	7,030	6,911	7,099	6,805	− 7%	0.024
ICU beds in hospitals with ICU but without IMCU	4,961	4,969	5,146	5,233	5,263	5,570	5,282	6%	0.004
ICU beds in hospitals with both ICU and IMCU	2,373	2,140	1,913	1,797	1,648	1,529	1,523	− 36%	0.002
Number of unit beds per 100,000 population									
IMCU beds	8.5	9.0	9.4	10.0	10.3	11.8	11.4	34%	0.004
IMCU beds in hospitals with IMCU but without ICU	2.7	3.0	3.2	3.5	3.6	4.2	4.3	62%	0.002
IMCU beds in hospitals with both IMCU and ICU	5.8	6.0	6.3	6.5	6.7	7.6	7.0	22%	0.004
ICU beds	5.8	5.6	5.6	5.6	5.5	5.7	5.4	− 6%	0.051
ICU beds in hospitals with ICU but without IMCU	3.9	3.9	4.1	4.1	4.2	4.4	4.2	8%	0.004
ICU beds in hospitals with both ICU and IMCU	1.9	1.7	1.5	1.4	1.3	1.2	1.2	− 35%	0.004
Number of hospitals									
Hospitals with IMCUs	703	751	780	810	822	867	856	22%	0.004
Hospitals with IMCU but without ICU	321	362	383	406	420	442	457	42%	0.002
Hospitals with both IMCU and ICU	382	389	397	404	402	425	399	4%	0.051
Hospitals with ICUs	655	632	620	614	597	608	577	− 12%	0.004
Hospitals with ICU but without IMCU	273	243	223	210	195	183	178	− 35%	0.002
Hospitals with both ICU and IMCU	382	389	397	404	402	425	399	4%	0.051

IMCU, intensive care unit; ICU, intermediate care unit

*p for trend was calculated using the Jonckheere–Terpstra trend test

**Fig. 1:**
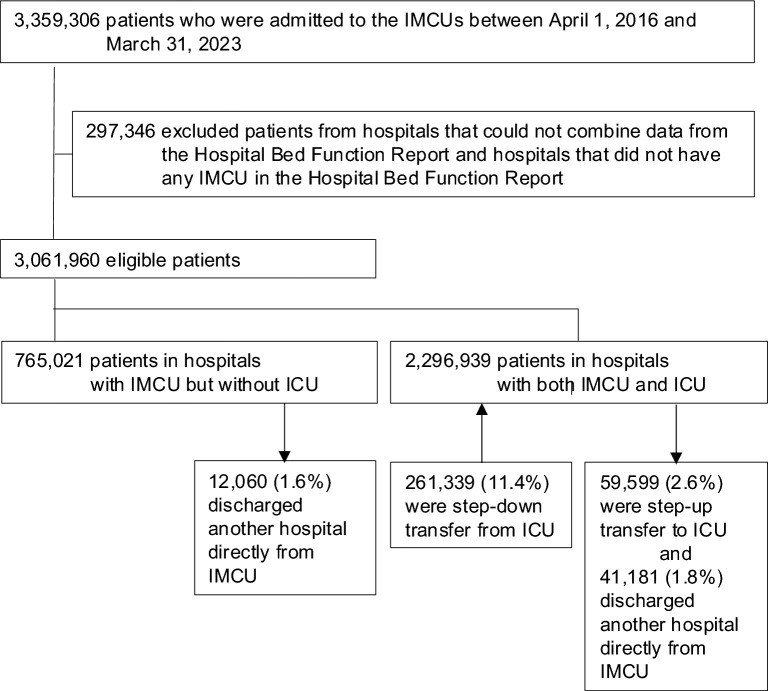
Patient flow chart for the analysis of the Diagnosis Procedure Combination database. IMCU, intensive care unit; ICU, intermediate care unit

In the analysis of the DPC database, after accounting for the inclusion criteria, we identified 3,061,960 eligible patients who were admitted to IMCUs from April 1, 2016 to March 31, 2023 (see Fig. 1). Of these, 765,021 (25%) were in hospitals with IMCUs but without ICUs and 2,296,939 (75%) were in hospitals with both IMCUs and ICUs. This cohort included 69% (7,451/10,736 beds) and 78% (5,707/7,334 beds) of all IMCU and ICU beds, respectively, in 2016, and 60% (8,447/14,185 beds) and 71% (4,811/6,805 beds) of all IMCU and ICU beds, respectively, in 2022 (see Supplementary Table 2, Additional File 1). In the 2016 analyses of eligible hospitals in the DPC database, hospitals with IMCUs but without ICUs were more likely to have fewer IMCU beds, total hospital beds, and ambulances received, were less likely to be academic hospitals and tertiary emergency hospitals, and had fewer hospital volume of patients in IMCUs compared to hospitals with both IMCUs and ICUs (see Supplementary Table 3, Additional File 1). There was no significant difference in the IMCU bed occupancy between the two groups. Similar findings were observed in the fiscal year 2022.

Of the 3,061,960 eligible patients, the mean age was 70.5 years (SD, 17.5), and 57% were male (Table 2). Compared with patients in hospitals with both IMCUs and ICUs, those in hospitals with IMCUs but without ICUs were more likely to be older, have alert consciousness, be admitted for elective and emergency surgery, be admitted to the IMCU on the day after admission, be admitted for cancer, receive dopamine on the day of IMCU admission, and less likely to be admitted for post-cardiac arrest.

**Table 2:** Baseline characteristics of patients in IMCUs in hospitals with and without ICUs

	Overall N = 3,061,960	Patients in hospitals with IMCU but without ICU N = 765,021	Patients in hospitals with both IMCU and ICU N = 2,296,939	SMD
Baseline characteristics				
Age, years	70.5 (17.5)	72.1 (16.1)	70.0 (17.9)	− 12
Male	1,745,888 (57.0)	424,621 (55.5)	1,321,267 (57.5)	4
Charlson Comorbidity Index	1.1 (1.5)	1.2 (1.5)	1.1 (1.5)	− 6
Japan Coma Scale at admission				
Alert	1,844,003 (60.2)	504,146 (65.9)	1,339,857 (58.3)	− 16
Dizziness	679,761 (22.2)	155,391 (20.3)	524,370 (22.8)	6
Somnolence	220,893 (7.2)	48,254 (6.3)	172,639 (7.5)	5
Coma	317,303 (10.4)	57,230 (7.5)	260,073 (11.3)	13
Location before hospitalization				
Home	2,695,240 (88.0)	674,870 (88.2)	2,020,370 (88.0)	− 1
Another hospital	171,706 (5.6)	32,126 (4.2)	139,580 (6.1)	9
Nursing home	195,014 (6.4)	58,025 (7.6)	136,989 (6.0)	− 6
Admission classification				
Elective surgery	489,145 (16.0)	179,816 (23.5)	309,329 (13.5)	− 26
Emergency surgery	336,332 (11.0)	109,877 (14.4)	226,455 (9.9)	− 14
Non- surgery	2,236,483 (73.0)	475,328 (62.1)	1,761,155 (76.7)	32
Length of hospital stay before IMCU				
On admission (day 1)	2,071,895 (67.7)	479,704 (62.7)	1,592,191 (69.3)	14
Day 2	342,534 (11.2)	110,799 (14.5)	231,735 (10.1)	− 13
Day 3–6	395,384 (12.9)	107,186 (14.0)	288,198 (12.5)	− 4
Day ≥ 7	252,147 (8.2)	67,332 (8.8)	184,815 (8.0)	− 3
Main etiologies for admission				
Stroke	566,065 (18.5)	158,988 (20.8)	407,077 (17.7)	− 8
Cancer	409,793 (13.4)	128,554 (16.8)	281,239 (12.2)	− 13
Acute abdominal diseases	304,325 (9.9)	82,291 (10.8)	222,034 (9.7)	− 4
Trauma	274,341 (9.0)	53,872 (7.0)	220,469 (9.6)	9
Acute heart failure	226,268 (7.4)	52,650 (6.9)	173,618 (7.6)	3
Acute coronary syndrome	195,603 (6.4)	55,065 (7.2)	140,538 (6.1)	− 4
Post cardiac arrest	109,334 (3.6)	14,613 (1.9)	94,721 (4.1)	13
Aortic dissection or aneurysm	97,927 (3.2)	20,364 (2.7)	77,563 (3.4)	4
Pneumonia	95,877 (3.1)	23,587 (3.1)	72,290 (3.1)	0
Aspiration	90,216 (2.9)	22,948 (3.0)	67,268 (2.9)	0
Sepsis	79,048 (2.6)	17,616 (2.3)	61,432 (2.7)	2
Procedures at IMCU admission*				
Invasive mechanical ventilation	298,965 (9.8)	67,785 (8.9)	231,180 (10.1)	4
Nasal high flow	23,440 (0.8)	3,931 (0.5)	19,509 (0.8)	4
Non-invasive mechanical ventilation	19,428 (0.6)	3,403 (0.4)	16,025 (0.7)	3
Red blood cell transfusion	175,421 (5.7)	50,507 (6.6)	124,914 (5.4)	− 5
Fresh frozen plasma transfusion	57,527 (1.9)	19,575 (2.6)	37,952 (1.7)	− 6
Platelet transfusion	20,922 (0.7)	8,033 (1.1)	12,889 (0.6)	− 5
Noradrenaline	204,259 (6.7)	61,934 (8.1)	142,325 (6.2)	− 7
Dopamine	112,954 (3.7)	48,664 (6.4)	64,290 (2.8)	− 17
Dobutamine	65,390 (2.1)	18,410 (2.4)	46,980 (2.0)	− 2
Adrenaline	60,362 (2.0)	18,969 (2.5)	41,393 (1.8)	− 5
Vasopressin	11,475 (0.4)	2,411 (0.3)	9,064 (0.4)	1
Cardiopulmonary resuscitation	116,771 (3.8)	20,884 (2.7)	95,887 (4.2)	8
Mechanical circulatory support	14,348 (0.5)	5,863 (0.8)	8,485 (0.4)	− 5
Renal replacement therapy	50,314 (1.6)	13,020 (1.7)	37,294 (1.6)	− 1

Continuous variables are summarized as means with standard deviations or medians with interquartile ranges, as appropriate. Categorical variables are expressed as frequencies and percentages

IMCU, intermediate care unit; ICU, intensive care unit; SMD, standardized mean difference

Regarding process measurements, the overall proportion of patients transferred between IMCU and ICU was 10.5% (Table 3). Of these, 81.4% (n = 261,339/320,938) were step-down transfer from ICU to IMCU, and 18.6% (n = 59,599/320,938) were step-up transfer from IMCU to ICU. Characteristics on the day of transfer between the ICU and IMCU are presented in Supplementary Table 4, Additional File 1. Life-sustaining therapies during IMCU stay were performed in 23.4% and 21.9% of patients in hospitals without and with ICUs, respectively, with no significant differences between the two groups. Among patients who received life-sustaining therapies during IMCU stay, 4.2% and 0.0% underwent step-up transfer from the IMCU to ICU in hospitals with and without ICUs, respectively, while 1.9% and 1.5% underwent hospital transfer directly from the IMCU in hospitals without and with ICUs, respectively, with no significant differences between the two groups (see Supplementary Table 5, Additional File 1).

**Table 3:** Process measurements and outcomes of patients admitted in IMCUs in hospitals with and without ICUs

Variables	Overall N = 3,061,960	Patients in hospitals with IMCU but without ICU N = 765,021	Patients in hospitals with both IMCU and ICU N = 2,296,939	SMD
Process measurements				
Transfer between IMCU and ICU	320,938 (10.5)	0 (0.0)	320,938 (14.0)	–
Step- down transfer from ICU to IMCU	261,339 (8.5)	0 (0.0)	261,339 (11.4)	–
Step-up transfer from IMCU to ICU	59,599 (1.9)	0 (0.0)	59,599 (2.6)	–
Hospital transfer directly from IMCU	53,241 (1.7)	12,060 (1.6)	41,181 (1.8)	2
Life-sustaining therapies during IMCU stay				
Any life-sustaining therapy	683,230 (22.3)	179,335 (23.4)	503,895 (21.9)	-4
Invasive mechanical ventilation	390,134 (12.7)	102,612 (13.4)	287,522 (12.5)	− 3
Noradrenaline	260,155 (8.5)	78,339 (10.2)	181,816 (7.9)	− 8
Adrenaline	71,499 (2.3)	21,675 (2.8)	49,824 (2.2)	− 4
Cardiopulmonary resuscitation	125,639 (4.1)	24,830 (3.2)	100,809 (4.4)	6
Mechanical circulatory support	18,644 (0.6)	7,763 (1.0)	10,881 (0.5)	− 6
Renal replacement therapy	100,181 (3.3)	25,118 (3.3)	75,063 (3.3)	0
Outcomes				
In- hospital mortality	364,313 (11.9)	86,739 (11.3)	277,574 (12.1)	2
IMCU mortality	221,483 (7.2)	48,248 (6.3)	173,235 (7.5)	5
Discharge destination				
Home	1,863,272 (60.9)	496,814 (64.9)	1,366,458 (59.5)	− 11
Another hospital	697,800 (22.8)	132,960 (17.4)	564,840 (24.6)	18
Nursing home	136,575 (4.5)	48,508 (6.3)	88,067 (3.8)	− 11
Length of IMCU stay, days	2.0 (1.0–5.0)	2.0 (1.0–5.0)	2.0 (1.0–4.0)	− 7
Length of hospital stay, days	15.0 (8.0–27.0)	17.0 (10.0–32.0)	14.0 (8.0–26.0)	− 11
Hospitalization costs, million yen	1.3 (0.7–2.1)	1.4 (0.8–2.2)	1.2 (0.7–2.0)	− 2

Continuous variables are summarized as means with standard deviations or medians with interquartile ranges, as appropriate. Categorical variables are expressed as frequencies and percentages

IMCU, intermediate care unit; ICU, intensive care unit; SMD, standardized mean difference

Among 296 hospitals equipped with IMCUs and ICUs in 2016, the median percentage of transfer between IMCU and ICU was 13.4% (interquartile range, 5.3%–24.2%), with significant variation between hospitals (see Supplementary Fig. 1, Additional File 1). The percentage of step-down transfer was 10.3% (2.5% to 20.3%), with significant variation between hospitals, whereas the percentage of step-up transfer was 2.6% (1.7% to 3.5%), with little variation among hospitals. Similar findings were observed for 2022 (see Supplementary Fig. 2, Additional File 1).

Regarding the outcomes, the crude in-hospital mortality was 11.3% and 12.1% in patients in hospitals with IMCUs but without ICUs and in those with both IMCUs and ICUs, respectively (Table 3). Patients in hospitals with IMCUs but without ICUs were more likely to be discharged to home or nursing homes and had longer hospital stays. There were no significant differences in the crude length of the IMCU stay or hospitalization costs. In hospitals with both IMCUs and ICUs, the in-hospital mortality was 12.3%, 8.5%, and 20.2% in patients who required no transfer, step-down transfer, and step-up transfer between IMCU and ICU, respectively (see Supplementary Table 6, Additional File 1).

In the patient-level trend analyses of the DPC database, the proportion of patients transferred between the IMCU and ICU decreased from 12.2% in 2016 to 9.3% in 2022 (Table 4). The proportion of step-down transfer from the ICU to the IMCU also decreased, whereas that of step-up transfer from the IMCU to the ICU did not change. The proportions of life-sustaining therapies during IMCU stay, in-hospital mortality, and IMCU mortality gradually increased during the study period.

**Table 4:** Trend analysis of the process measurements and outcomes of patients in the IMCU from 2016 to 2022

	Fiscal year							
	2016	2017	2018	2019	2020	2021	2022	p for
	N = 382,520	N = 441,270	N = 420,324	N = 450,411	N = 435,445	N = 485,343	N = 446,647	Trend*
Process measurements								
Transfer between IMCU and ICU	46,728 (12.2)	49,535 (11.2)	46,500 (11.1)	48,935 (10.9)	43,923 (10.1)	43,990 (9.1)	41,327 (9.3)	< 0.001
Step-down transfer from ICU to IMCU	39,121 (10.2)	41,050 (9.3)	38,590 (9.2)	40,204 (8.9)	34,955 (8.0)	34,507 (7.1)	32,912 (7.4)	< 0.001
Step-up transfer from IMCU to ICU	7,607 (2.0)	8,485 (1.9)	7,910 (1.9)	8,731 (1.9)	8,968 (2.1)	9,483 (2.0)	8,415 (1.9)	0.56
Hospital transfer directly from IMCU	6,605 (1.7)	6,836 (1.5)	6,517 (1.6)	6,719 (1.5)	7,920 (1.8)	9,915 (2.0)	8,729 (2.0)	< 0.001
Life-sustaining therapies during IMCU	80,480 (21.0)	96,048 (21.8)	93,374 (22.2)	100,551 (22.3)	97,354 (22.4)	110,733 (22.8)	104,690 (23.4)	< 0.001
Outcomes								
In-hospital mortality	44,967 (11.8)	52,067 (11.8)	48,885 (11.6)	52,178 (11.6)	51,622 (11.9)	58,441 (12.0)	56,153 (12.6)	< 0.001
IMCU mortality	27,063 (7.1)	31,297 (7.1)	29,348 (7.0)	30,928 (6.9)	31,793 (7.3)	36,154 (7.4)	34,900 (7.8)	< 0.001
Discharge destination								
Home	231,593 (60.5)	267,860 (60.7)	257,749 (61.3)	274,719 (61.0)	263,105 (60.4)	298,018 (61.4)	270,228 (60.5)	0.32
Another hospital	89,149 (23.3)	102,116 (23.1)	95,964 (22.8)	103,826 (23.1)	101,150 (23.2)	106,844 (22.0)	98,751 (22.1)	< 0.001
Nursing home	16,811 (4.4)	19,227 (4.4)	17,726 (4.2)	19,688 (4.4)	19,568 (4.5)	22,040 (4.5)	21,515 (4.8)	< 0.001
Length of IMCU stay, days	2.0 (1.0–4.0)	2.0 (1.0–4.0)	2.0 (1.0–4.0)	2.0 (1.0–4.0)	2.0 (1.0–5.0)	2.0 (1.0–5.0)	2.0 (1.0–5.0)	< 0.001
Length of hospital stay, days	16.0 (8.0–29.0)	16.0 (8.0–29.0)	15.0 (8.0–28.0)	15.0 (8.0–28.0)	15.0 (8.0–27.0)	14.0 (8.0–26.0)	14.0 (8.0–27.0)	< 0.001
Hospitalization costs, million yen	1.2 (0.7–2.0)	1.2 (0.7–2.0)	1.2 (0.7–2.1)	1.3 (0.7–2.1)	1.3 (0.7–2.1)	1.3 (0.7–2.1)	1.3 (0.8–2.2)	< 0.001

Continuous variables are summarized as medians with interquartile ranges. Categorical variables are expressed as frequencies and percentages

IMCU, intermediate care unit; ICU, intensive care unit; SMD, standardized mean difference

*p for trend was calculated using the Jonckheere–Terpstra trend test for continuous variables and the Cochran–Armitage trend test for binomial proportions

After adjusting for potential confounders, in-hospital mortality for patients in hospitals with IMCUs but without ICUs was significantly higher than that in hospitals with both IMCUs and ICUs (adjusted odds ratio: 1.15; 95% CI: 1.10–1.20; *p* < 0.001; Table 5). In the subgroup analysis for patients aged ≥ 75 or < 75 years, no heterogeneity was observed in the effect of IMCU admission in hospitals without ICU on in-hospital mortality between the groups (p for interaction = 0.829). For hospitals without ICU, the adjusted odds ratio for IMCU admission and in-hospital mortality was 1.30 (95% CI: 1.23–1.37) and 1.12 (95% CI: 1.06–1.18) for patients with and without life-sustaining therapies, respectively, with significant heterogeneity in the effect of IMCU admission on in-hospital mortality between the groups (p for interaction < 0.001). In the sensitivity analyses for categorizing percentage of hospital transfer between IMCU and ICU into quartiles, patients in hospitals with both IMCUs and ICUs had significantly lower in-hospital mortality rates as the percentage of hospital transfer between IMCU and ICU exceeded 10.8%. The adjusted odds ratios for in-hospital mortality in the 10.8%–20.0% and 20.0%–95.1% hospital transfer groups were 0.90 (95% CI: 0.88–0.93) and 0.83 (95% CI: 0.80–0.86), respectively, with the 0%–4.9% hospital transfer group as reference. One-to-one propensity score matching showed 762,882 matched pairs. After matching, in-hospital mortality for patients in hospitals with IMCUs but without ICUs remained significantly higher than that in hospitals with both IMCUs and ICUs (adjusted odds ratio: 1.23; 95% CI: 1.10–1.20). In the sensitivity analysis excluding 187,621 patients who died without receiving invasive mechanical ventilation, the results were similar to the main analysis and the point estimates and CI were further increased (adjusted odds ratio: 1.49; 95% CI: 1.40–1.58).

**Table 5:** Results of the multilevel mixed-effects regression models for the association between IMCU admission in hospitals with and without ICUs and in-hospital mortality

	In-hospital mortality	Odds ratio (95% CIs)	p value
Main analysis			
Patients in hospitals with IMCU but without ICU	86,739/765,021 (11.3%)	1.15 (1.10–1.20)	< 0.001
Patients in hospitals with both IMCU and ICU	277,574/2,296,939 (12.1%)	Ref	–
Subgroup analysis			
1. Patients aged ≥ 75 or < 75 years			0.358*
Patients aged ≥ 75 years			
Patients in hospitals with IMCU but without ICU	61,990/393,066 (15.8%)	1.19 (1.14–1.25)	< 0.001
Patients in hospitals with both IMCU and ICU	183,572/1,110,267 (16.5%)	Ref	–
Patients aged < 75 years			
Patients in hospitals with IMCU but without ICU	24,749/371,955 (6.7%)	1.16 (1.10–1.23)	< 0.001
Patients in hospitals with both IMCU and ICU	94,002/1,186,672 (7.9%)	Ref	–
2. Life-sustaining therapies (LSTs) during IMCU			< 0.001*
Patients who required LSTs during IMCU			
Patients in hospitals with IMCU but without ICU	52,265/179,335 (29.1%)	1.30 (1.23–1.37)	< 0.001
Patients in hospitals with both IMCU and ICU	173,521/503,895 (34.4%)	Ref	–
Patients who did not require LSTs during IMCU			
Patients in hospitals with IMCU but without ICU	34,474/585,686 (5.9%)	1.12 (1.06–1.18)	< 0.001
Patients in hospitals with both IMCU and ICU	104,053/1,793,044 (5.8%)	Ref	–
Sensitivity analysis			
1. Categorizing hospital transfer rate into quartiles			
Patients in hospitals with IMCU but without ICU	86,739/765,021 (11.3%)	1.09 (1.04–1.14)	< 0.001
Patients in hospitals with both IMCU and ICU			
Hospital transfer rate between IMCUs and ICUs			
0%–4.9%	99,867/576,354 (17.3%)	Ref	–
4.9%–10.8%	66,156/572,882 (11.6%)	1.00 (0.98–1.03)	0.80
10.8%–20.0%	60,219/573,820 (10.5%)	0.90 (0.88–0.93)	< 0.001
20.0%–95.1%	51,332/573,883 (8.9%)	0.83 (0.80–0.86)	< 0.001
2. Propensity score matching analysis			
Patients in hospitals with IMCU but without ICU	86,429/762,882 (11.3%)	1.23 (1.16–1.30)	< 0.001
Patients in hospitals with both IMCU and ICU	83,440/762,882 (10.9%)	Ref	–
3. Excluding patients who died without IMV			
Patients in hospitals with IMCU but without ICU	40,823/719,105 (5.7%)	1.49 (1.40–1.58)	< 0.001
Patients in hospitals with both IMCU and ICU	135,869/2,155,234 (6.3%)	Ref	–

The covariates included fiscal year, age, sex, Charlson Comorbidity Index, Japan Coma Scale score at admission, location before hospitalization, admission classification, length of hospital stay before IMCU admission, main etiology of admission, and organ support therapy at IMCU admission

IMCU, intermediate care unit; ICU, intensive care unit; CI, confidence interval; LSTs, life-sustaining therapies; IMV, invasive mechanical ventilation

Hospitalization costs for patients in hospitals with IMCUs but without ICUs was significantly higher than those in hospitals with both IMCUs and ICUs (relative risk: 1.05; 95% CI: 1.04–1.06; *p* < 0.001) (see Supplementary Table 7, Additional File 1).

## Discussion

This study described the process and outcome measurements of IMCUs in hospitals with and without ICUs in Japan at the national level from 2016 to 2022.

Based on an analysis of the Hospital Bed Function Report, there has been a marked increase in the number of IMCU beds in Japan, which is particularly evident for IMCU in hospitals without ICUs, wherein the number of beds significantly increased between 2016 and 2022, accounting for 38% of all IMCU beds. Furthermore, the percentage of patients transferred between IMCUs and ICUs has consistently decreased each year.

In Japan, there are no government regulations mandating that IMCUs be built in hospitals with ICUs, and there are no regional target bed numbers for IMCUs or ICUs. This leaves decisions regarding the plan to build IMCUs and ICUs entirely up to the hospitals, which contributes to the current trend and regional disparities in critical care resources in Japan [19–21]. Given the reduction in human resources and costs associated with building IMCUs compared to ICUs, it is not surprising that the number of IMCUs in hospitals without ICUs is increasing. This increase in IMCU beds is a global trend, which suggests that the increase in hospitals with IMCUs but without ICUs in Japan may also occur internationally [5].

The decrease in step-down transfers from the ICU to IMCU may be attributed to the reduced number of IMCU beds in hospitals with ICUs. Conversely, the unchanged proportion of step-up transfers from the IMCU to ICU over the study period may reflect increasing patient severity, counterbalancing the impact of fewer IMCU beds. Additionally, step-down transfers may be more adaptable to changes in ICU bed availability, whereas step-up transfers, driven by patient deterioration, are less flexible and hence showed minimal variability.

Our results showed that in hospitals with IMCUs but without ICUs, as in hospitals with both IMCUs and ICUs, about one in four patients required life-sustaining therapies during IMCU stay, with a mortality rate of approximately 12%. This finding is contrary to the recommendation in the ICU admission guidelines of both Japan and Western countries, wherein patients requiring life-sustaining therapies should first be treated in the ICU [1–4]. Although IMCU may be optimal for patients who require mostly monitoring with expected morality low, it is difficult to precisely predict which patients will deteriorate and require step-up transfer from the IMCU to ICU for more intensive treatment at the time of IMCU admission [22–24], and the occurrence of such cases is inevitable. Furthermore, transferring all IMCU patients who need life-sustaining therapies from a hospital without ICUs to one with ICUs may not be practical owing to the large number of such patients. Therefore, providing an optimal level of critical care for patients in hospitals with IMCUs, but not ICUs, is challenging, and the need for step-up ICUs in the same hospital have become more necessary.

Patients in hospitals with IMCUs but without ICUs exhibited higher in-hospital mortality and hospitalization costs than those in hospitals with both IMCUs and ICUs. The increased mortality in hospitals without ICU was more pronounced in patients requiring life-sustaining therapies, whereas no such effect was observed in patients admitted mainly for monitoring without life-sustaining therapies. Previous studies have shown that treating critically ill patients requiring organ support therapies in IMCUs instead of ICUs lead to worse outcomes in patients with invasive mechanical ventilation [25, 26], mechanical circulatory support [27, 28], and septic shock [29, 30]. Therefore, the results of this study may be due to patients requiring life-sustaining therapies not being treated at the appropriate level of care units, which is consistent with previous studies; thus, hospitals with IMCUs but without ICUs are likely to violate the core concept of IMCUs of “without worsening the health outcomes of patients admitted to IMCUs” [5]. Another contributing factor could be the lack of support by intensivists and ICU nurses for patients in IMCU beds. Our findings recommend that IMCUs be placed in hospitals with ICUs. Considering the recent increasing trend of hospitals with IMCUs but without ICUs, a mandatory regulation for IMCUs to be established along with ICUs in the same hospital is desirable to improve outcomes for critically ill patients.

Even among hospitals with both IMCUs and ICUs, considerable heterogeneity was observed in the percentage of patients transferred between them. This study showed that hospitals with a higher proportion of transfer between IMCU and ICU had better in-hospital mortality rates, especially when the proportion exceeded 10%, which suggests that hospitals with a more flexible transfer system between IMCU and ICU that ensures appropriate level of care for patients according to their severity, are better equipped to improve patient survival. A previous study reported that 18.8% of ICU patients were discharged to the IMCU [31], suggesting that transfer between IMCU and ICU are relatively low in Japan. Therefore, it is desirable to develop a healthcare system and structure that facilitates intrahospital transfer between IMCU and ICU.

Our findings highlight an increasing trend in the number of hospitals with IMCUs but without ICUs in Japan, which may result in a lack of appropriate levels of care and worsening patient outcomes. Policymakers may need to reconsider the current critical care system to address these disparities and trends, and ensure that severely ill patients receive the appropriate level of care they need. Heterogeneity in transfer practices among hospitals with both IMCU and ICU underscores the importance of implementing protocols or guidelines for patient management in IMCUs and ICUs.

The strength of this study lies in its use of a large nationwide inpatient database, which provides a nationally representative cohort of a large number of IMCU and ICU patients with long-term trends. To the best of our knowledge, this is the first study to assess the processes and outcomes of IMCUs in hospitals with and without ICU.

The present study has several limitations. First, the observational nature of the study design prevented us from drawing causal conclusions. Second, factors that were not measured at the patient and hospital levels may have influenced the results. Third, the definition, organization, staffing, equipment, interventions, patient case-mix, and utilization of IMCUs varies between countries, influencing patient case-mix and outcomes [5, 32]. Therefore, as this population-based study in Japan reflects findings from a single country, the results may not be generalizable to other countries. Future studies should address these limitations and validate our study findings.

## Conclusions

IMCUs in hospitals without ICUs became increasingly common in Japan, and patient care in these hospitals was associated with worse patient outcomes. The findings suggested that IMCUs might be better placed in hospitals with ICUs and emphasized the potential need for a better critical care delivery system that could provide an appropriate level of care according to patient severity, with enhanced transfer between IMCU and ICU. However, these findings should be interpreted within the context of local healthcare systems. Further studies are needed to evaluate the external validity of these results across different countries and institutions.

## Supplementary Information

Additional file1 (PDF 418 KB)

## Abbreviations

ICU: Intensive care unit
IMCU: Intermediate care unit
DPC: Diagnosis procedure combination
SD: Standard deviation
SMD: Standardized mean differences
CI: Confidence interval

## Declarations

### Availability of data and materials

The dataset analyzed in the current study is not publicly available because of contracts with the hospitals that provided the data to the database.
