# Supplementary material for: In-hospital mortality of patients admitted to the intermediate care unit in hospitals with and without an intensive care unit: a nationwide inpatient database study
Source: Crit Care. 2025 Jan 20;29:34. doi: 10.1186/s13054-025-05275-0 (PMC11744974; doi:10.1186/s13054-025-05275-0)

Additional File 1

In-hospital mortality of patients admitted to the intermediate care unit in hospitals with and without an intensive care unit: a nationwide inpatient database study

Hiroyuki Ohbe[1,2*], Daisuke Kudo[1,3], Yuya Kimura[4], Hiroki Matsui[2], Hideo Yasunaga[2], Shigeki Kushimoto[1,3]

[1]Department of Emergency and Critical Care Medicine, Tohoku University Hospital, 1-1 Seiryo-machi, Aoba-ku, Sendai 980-8574, Japan

[2]Department of Clinical Epidemiology and Health Economics, School of Public Health, The University of Tokyo, 7-3-1 Hongo, Bunkyo-ku, Tokyo 113-0033, Japan

[3]Division of Emergency and Critical Care Medicine, Tohoku University Graduate School of Medicine, 2-1 Seiryo-machi, Aoba-ku, Sendai, 980-8575, Japan

[4]Department of Health Services Research, Graduate School of Medicine, The University of Tokyo, Tokyo, 7-3-1 Hongo, Bunkyo-ku, Tokyo 113-0033, Japan

List of additional file 1

Supplementary Table 1. Japanese medical procedure codes used to define ICUs and IMCUs in 2022.

Supplementary Table 2. Eligible units and hospitals that successfully combined the Hospital Bed Function Report and DPC databases.

Supplementary Table 3. Comparison of hospital characteristics between hospitals with and without ICUs in fiscal years 2016 and 2022.

Supplementary Table 4. Characteristics on the day of transfer between ICU and IMCU.

Supplementary Table 5. Comparison of process measurements and outcomes between patients admitted in IMCUs in hospitals with and without ICUs and patients with and without life-sustaining therapies during IMCU.

Supplementary Table 6. Outcomes of patients admitted in IMCUs in hospitals with ICUs stratified by transfer between IMCU and ICU.

Supplementary Table 7. Results of the multilevel mixed-effects regression models for the association between IMCU admission in hospitals with and without ICUs and hospitalization costs.

Supplementary Figure 1. Transfers between IMCUs and ICUs at the hospital level in fiscal year 2016.

Supplementary Figure 2. Transfers between IMCUs and ICUs at the hospital level in fiscal year 2022.

Supplementary Table 1. Japanese medical procedure codes used to define ICUs and IMCUs in 2022.

Type	Code	Description	Cost per day, yen	Intensivist	Non-intensivist	Physician night shift	Nurse to patient ratio	ICU Nurse	Clinical Engineer
ICU	A3011	ICU management fee 1	142,110**	≥2 in ICU 24/7	–	Dedicated to ICU	1:2	Required ***	24/7 in hospital
ICU	A3012	ICU management fee 2	142,110**	≥2 in ICU 24/7	–	Dedicated to ICU	1:2	Required ***	24/7 in hospital
ICU	A3013	ICU management fee 3	96,970**	–	≥1 in ICU 24/7	Dedicated to ICU	1:2	–	–
ICU	A3014	ICU management fee 4	96,970**	–	≥1 in ICU 24/7	Dedicated to ICU	1:2	–	–
ICU	A3002	Emergency and critical care unit management fee 2	118,020*	–	≥1 in ICU 24/7	Dedicated to ICU	1:2	–	–
ICU	A3004	Emergency and critical care unit management fee 4	118,020*	–	≥1 in ICU 24/7	Dedicated to ICU	1:2	–	–
ICU	A301-4	Pediatric ICU management fee	163,170**	≥2 in ICU 24/7	–	Dedicated to ICU	1:2	–	–
IMCU	A3001	Emergency and critical care unit management fee 1	102,230*	–	≥1 in IMCU 24/7	Dedicated to IMCU	1:4	–	–
IMCU	A3003	Emergency and critical care unit management fee 3	102,230*	–	≥1 in IMCU 24/7	Dedicated to IMCU	1:4	–	–

IMCU	A301-21	High care unit management fee 1	68,550	–	≥1 in hospital 24/7	On-call permit	1:4	–	–
IMCU	A301-22	High care unit management fee 2	42,240	–	≥1 in hospital 24/7	On-call permit	1:5	–	–
IMCU	A301-3	Stroke care unit management fee	60,130	–	Neurologist ≥1 in hospital 24/7	On-call permit	1:3	–	–

*Cost per day for the first three days after admission in 2022. **Cost per day for the first seven days after admission in 2022. ***A full-time ICU nurse work for at least 20 hours per week in the ICU. ICU, intensive care unit; IMCU, intermediate care unit.

Supplementary Table 2. Eligible units and hospitals that successfully combined the Hospital Bed Function Report and DPC databases.

	Fiscal year						
	2016	2017	2018	2019	2020	2021	2022
**Number of beds combined with the DPC database**							
Total number of IMCU beds	7,451	8,355	7,882	8,341	8,354	9,501	8,447
IMCU beds in hospitals with IMCU but without ICU	1,595	2,152	2,008	2,223	2,188	2,551	2,233
IMCU beds in hospitals with both IMCU and ICU	5,856	6,203	5,874	6,118	6,166	6,950	6,214
Total number of ICU beds	5,707	5,617	5,208	5,200	5,021	5,261	4,811
ICU beds in hospitals with ICU but without IMCU	4,017	4,069	3,969	4,074	4,048	4,315	3,921
ICU beds in hospitals with both ICU and IMCU	1,690	1,548	1,239	1,126	973	946	890
**Number of hospitals combined with the DPC database**							
Hospitals with IMCU	452	504	473	492	479	516	462
Hospitals with IMCU but without ICU	156	198	187	199	193	214	187
Hospitals with both IMCU and ICU	296	306	286	293	286	302	275
Hospitals with ICU	474	466	420	417	390	404	365
Hospitals with ICU but without IMCU	178	160	134	124	104	102	90
Hospitals with both ICU and IMCU	296	306	286	293	286	302	275
**Percentage of units combined with the DPC database**							
Total number of IMCU beds	69%	73%	66%	66%	64%	64%	60%
IMCU beds in hospitals with IMCU but without ICU	47%	57%	50%	50%	48%	48%	41%
IMCU beds in hospitals with both IMCU and ICU	80%	81%	74%	75%	73%	73%	71%
Total number of ICU beds	78%	79%	74%	74%	73%	74%	71%
ICU beds in hospitals with ICU but without IMCU	81%	82%	77%	78%	77%	77%	74%
ICU beds in hospitals with both ICU and IMCU	71%	72%	65%	63%	59%	62%	58%
**Percentage of hospitals combined with the DPC database**							
Hospitals with IMCU	64%	67%	61%	61%	58%	60%	54%
Hospitals with IMCU but without ICU	49%	55%	49%	49%	46%	48%	41%
Hospitals with both IMCU and ICU	77%	79%	72%	73%	71%	71%	69%
Hospitals with ICU	72%	74%	68%	68%	65%	66%	63%
Hospitals with ICU but without IMCU	65%	66%	60%	59%	53%	56%	51%
Hospitals with both ICU and IMCU	77%	79%	72%	73%	71%	71%	69%

DPC, diagnostic procedure combination; IMCU, intermediate care unit; ICU, intensive care unit.

Supplementary Table 3. Comparison of hospital characteristics between hospitals with and without ICUs in fiscal years 2016 and 2022.

	Fiscal year 2016			Fiscal year 2022		
Hospital characteristics	Hospitals with IMCU but without ICU N=156	Hospitals with both IMCU and ICU N=296	SMD	Hospitals with IMCU but without ICU N=187	Hospitals with both IMCU and ICU N=275	SMD
Hospitals with IMCU	156 (100.0)	296 (100.0)	–	187 (100.0)	275 (100.0)	–
Number of IMCU beds	8 (6–12)	18 (10–25)	90	9 (6–15)	20 (12–30)	89
Hospitals with ICU	–	296 (100.0)	–	–	275 (100.0)	–
Number of ICU beds	–	10 (7–16)	–	–	10 (8–18)	–
Number of total hospital beds	283 (199–364)	552 (400–682)	151	292 (215–360)	548 (413–676)	161
Academic hospital	0 (0.0)	59 (19.9)	70	0 (0.0)	63 (22.9)	77
Tertiary emergency hospital	20 (12.8)	184 (62.2)	118	23 (12.3)	180 (65.5)	130
Annual number of ambulances received	1,985 (1,250–3,057)	4,371 (2,698–6,076)	118	2,350 (1,472–3,334)	4,292 (2,941–6,021)	104
Annual hospital volume of IMCU patients	429 (288–691)	827 (482–1,337)	83	520 (366–787)	1,028 (656–1,576)	97
Reimbursement rates for IMCU bed, %*	54.7 (43.4–69.5)	55.3 (39.8–72.0)	-6	58.6 (44.6–71.8)	56.8 (40.3–73.4)	-1
IMCU bed occupancy, %**	64.0 (49.3–79.7)	80.3 (66.3–91.0)	60	62.5 (47.9–78.1)	69.5 (51.5–81.7)	15

Continuous variables are summarized as medians with interquartile ranges, and categorical variables are expressed as frequencies and percentages.

*Reimbursement rate for IMCU bed was calculated by dividing the total reimbursement days for IMCU in a year by the number of IMCU beds multiplied by 365. Under the National Health Insurance in Japan, reimbursement for IMCU is capped at 21 days per hospitalization, which excluded long-term IMCU stays

**IMCU bed occupancy was calculated by dividing the total occupied days from the Hospital Bed Function Report in a year by the number of IMCU beds multiplied by 365.

ICU, intensive care unit; IMCU, intermediate care unit; SMD, standardized mean difference.

Supplementary Table 4. Characteristics on the day of transfer between ICU and IMCU.

Characteristics	Characteristics on the day of step-down transfer from ICU to IMCU N=261,339	Characteristics on the day of step-up transfer from IMCU to ICU N=59,599
Age, years	68.5 (16.3)	69.7 (15.9)
Male	160,832 (61.5)	37,459 (62.9)
Charlson Comorbidity Index	1.2 (1.4)	1.4 (1.5)
Japan Coma Scale at admission		
Alertness	148,852 (57.0)	35,929 (60.3)
Dizziness	48,375 (18.5)	14,691 (24.6)
Somnolence	23,999 (9.2)	4,692 (7.9)
Coma	40,113 (15.3)	4,287 (7.2)
Location before hospitalization		
Home	227,603 (87.1)	50,944 (85.5)
Another hospital	25,390 (9.7)	6,707 (11.3)
Nursing home	8,346 (3.2)	1,948 (3.3)
Admission classification		
Elective surgery	348 (0.1)	3,775 (6.3)
Emergency surgery	11,741 (4.5)	29,819 (50.0)
Non-surgery	249,250 (95.4)	26,005 (43.6)
Length of hospital stay before IMCU		
On admission (day 1)	50,734 (19.4)	51,852 (87.0)
Day 2	113,315 (43.4)	2,151 (3.6)
Day 3-6	97,290 (37.2)	2,814 (4.7)
Day ≥7	43,356 (16.6)	2,782 (4.7)
Main etiologies for admission		
Stroke	27,357 (10.5)	9,872 (16.6)
Cancer	21,624 (8.3)	5,686 (9.5)
Acute abdominal diseases	27,129 (10.4)	6,602 (11.1)
Trauma	17,633 (6.7)	4,788 (8.0)
Acute heart failure	30,024 (11.5)	8,195 (13.8)
Acute coronary syndrome	7,759 (3.0)	6,591 (11.1)
Post cardiac arrest	25,356 (9.7)	900 (1.5)
Aortic dissection or aneurysm	6,478 (2.5)	3,938 (6.6)
Pneumonia	6,125 (2.3)	2,376 (4.0)
Aspiration	13,194 (5.0)	1,474 (2.5)
Sepsis	8,325 (3.2)	2,739 (4.6)
Organ support therapy at transfer		
Invasive mechanical ventilation	9,703 (3.7)	20,388 (34.2)
Nasal high flow	3,631 (1.4)	1,289 (2.2)
Non-invasive mechanical ventilation	41,652 (15.9)	772 (1.3)
Red blood cell transfusion	2,913 (1.1)	16,711 (28.0)
Fresh frozen plasma transfusion	1,325 (0.5)	11,185 (18.8)
Platelet transfusion	9,080 (3.5)	6,260 (10.5)
Noradrenaline	631 (0.2)	24,998 (41.9)
Dopamine	13,317 (5.1)	9,648 (16.2)
Dobutamine	12,326 (4.7)	10,498 (17.6)

Adrenaline	1,306 (0.5)	3,196 (5.4)
Vasopressin	169 (0.1)	2,914 (4.9)
Cardiopulmonary resuscitation	1,014 (0.4)	2,026 (3.4)
Mechanical circulatory support	9,698 (3.7)	3,351 (5.6)
Renal replacement therapy	22,107 (8.5)	6,536 (11.0)

Continuous variables are summarized as means with standard deviations or medians with interquartile ranges, as appropriate. Categorical variables are expressed as frequencies and percentages.

IMCU, intermediate care unit; ICU, intensive care unit; SMD, standardized mean difference.

Supplementary Table 5. Comparison of process measurements and outcomes between patients admitted in IMCUs in hospitals with and without ICUs and patients with and without life-sustaining therapies during IMCU.

	Patients with LSTs during IMCU stay			Patients without LSTs during IMCU stay		
Variables	Patients in hospitals with IMCU but without ICU N=179,335	Patients in hospitals with both IMCU and ICU N=503,895	SMD	Patients in hospitals with IMCU but without ICU N=585,686	Patients in hospitals with both IMCU and ICU N=1,793,044	SMD
**Process measurements**						
Transfer between IMCU and ICU	0 (0.0)	89,478 (17.8)	–	0 (0.0)	231,460 (12.9)	–
Step-down transfer from ICU to IMCU	0 (0.0)	68,246 (13.5)	–	0 (0.0)	193,093 (10.8)	–
Step-up transfer from IMCU to ICU	0 (0.0)	21,232 (4.2)	–	0 (0.0)	38,367 (2.1)	–
Hospital transfer directly from IMCU	3,420 (1.9)	7,498 (1.5)	-3	8,640 (1.5)	33,683 (1.9)	3
**Outcomes**						
In-hospital mortality	52,265 (29.1)	173,521 (34.4)	11	34,474 (5.9)	104,053 (5.8)	0
IMCU mortality	36,497 (20.4)	135,366 (26.9)	15	11,751 (2.0)	37,869 (2.1)	1
Discharge destination						
Home	88,055 (49.1)	201,000 (39.9)	-19	408,759 (69.8)	1,165,458 (65.0)	-10
Another hospital	31,743 (17.7)	117,425 (23.3)	14	101,217 (17.3)	447,415 (25.0)	19
Nursing home	7,272 (4.1)	11,949 (2.4)	-10	41,236 (7.0)	76,118 (4.2)	-12
Length of IMCU stay, days	3.0 (1.0-8.0)	2.0 (1.0-6.0)	-18	2.0 (1.0-4.0)	2.0 (1.0-4.0)	-1
Length of hospital stay, days	20.0 (9.0-40.0)	15.0 (4.0-31.0)	-12	17.0 (10.0-30.0)	14.0 (8.0-25.0)	-10
Hospitalization costs, million yen	1.7 (0.9-3.1)	1.4 (0.5-2.9)	-5	1.3 (0.8-2.0)	1.2 (0.7-1.9)	0

Continuous variables are summarized as means with standard deviations or medians with interquartile ranges, as appropriate. Categorical variables are expressed as frequencies and percentages.

IMCU, intermediate care unit; ICU, intensive care unit; SMD, standardized mean difference.

Supplementary Table 6. Outcomes of patients admitted in IMCUs in hospitals with ICUs stratified by transfer between IMCU and ICU.

Outcomes	Overall N=2,296,939	No transfer between IMCU and ICU N=1,976,001	Step-down transfer from ICU to IMCU N=261,339	Step-up transfer from IMCU to ICU N=59,599
In-hospital mortality	277,574 (12.1)	243,417 (12.3)	22,107 (8.5)	12,050 (20.2)
IMCU mortality	173,235 (7.5)	165,773 (8.4)	6,844 (2.6)	618 (1.0)
Discharge destination				
Home	1,366,458 (59.5)	1,198,533 (60.7)	142,111 (54.4)	25,814 (43.3)
Another hospital	564,840 (24.6)	452,811 (22.9)	91,528 (35.0)	20,501 (34.4)
Nursing home	88,067 (3.8)	81,240 (4.1)	5,593 (2.1)	1,234 (2.1)
Length of IMCU stay, days	2.0 (1.0-4.0)	2.0 (1.0-4.0)	3.0 (2.0-6.0)	3.0 (1.0-7.0)
Length of hospital stay, days	14.0 (8.0-26.0)	13.0 (7.0-23.0)	24.0 (15.0-42.0)	35.0 (21.0-57.0)
Hospitalization costs, million yen	1.2 (0.7-2.0)	1.1 (0.6-1.7)	2.8 (1.6-4.7)	3.6 (2.3-5.7)

Continuous variables are summarized as medians with interquartile ranges, as appropriate. Categorical variables are expressed as frequencies and percentages.

IMCU, intermediate care unit; ICU, intensive care unit; SMD, standardized mean difference.

Supplementary Table 7. Results of the multilevel mixed-effects regression models for the association between IMCU admission in hospitals with and without ICUs and hospitalization costs.

	Hospitalization costs, million yen	Relative risk (95% CIs)	p value
**Main analysis**			
Patients in hospitals with IMCU but without ICU	1.4 (0.8–2.2)	1.05 (1.04–1.06)	<0.001
Patients in hospitals with both IMCU and ICU	1.2 (0.7–2.0)	Ref.	–
**Subgroup analysis**			
1. Aged ≥75 or <75 years			0.829*
Aged ≥75 years			
Patients in hospitals with IMCU but without ICU	1.4 (0.8–2.2)	1.04 (1.03–1.05)	<0.001
Patients in hospitals with both IMCU and ICU	1.2 (0.7–2.0)	Ref.	–
Aged <75 years			
Patients in hospitals with IMCU but without ICU	1.3 (0.8–2.1)	1.04 (1.03–1.05)	<0.001
Patients in hospitals with both IMCU and ICU	1.2 (0.7–2.1)	Ref.	–
2. Life-sustaining therapies (LSTs) during IMCU			<0.001*
Patients who required LSTs during IMCU			
Patients in hospitals with IMCU but without ICU	1.7 (0.9–3.1)	1.00 (0.99–1.03)	0.301
Patients in hospitals with both IMCU and ICU	1.4 (0.5–2.9)	Ref.	–
Patients who did not require LSTs during IMCU			
Patients in hospitals with IMCU but without ICU	1.3 (0.8–2.0)	1.05 (1.04–1.06)	<0.001
Patients in hospitals with both IMCU and ICU	1.2 (0.7–1.9)	Ref.	–
**Sensitivity analysis**			
1. Categorizing hospital transfer rate into quartiles			
Patients in hospitals with IMCU but without ICU	1.4 (0.8–2.2)	1.05 (1.04–1.06)	<0.001
Patients in hospitals with both IMCU and ICU			
Hospital transfer rate between IMCUs and ICUs			
0%–4.9%	1.0 (0.6–1.7)	Ref.	–
4.9%–10.8%	1.2 (0.7–2.0)	1.00 (0.99–1.00)	0.023
10.8%–20.0%	1.2 (0.7–2.0)	1.01 (1.00–1.01)	0.003
20.0%–95.1%	1.4 (0.8–2.5)	1.03 (1.02–1.03)	<0.001
2. Propensity score matching analysis			
Patients in hospitals with IMCU but without ICU	1.4 (0.8–2.1)	0.88 (0.87–0.89)	<0.001
Patients in hospitals with both IMCU and ICU	1.5 (0.9–2.5)	Ref.	–
3. Excluding patients who died without IMV			
Patients in hospitals with IMCU but without ICU	1.4 (0.8–2.2)	1.06 (1.05–1.07)	<0.001
Patients in hospitals with both IMCU and ICU	1.2 (0.7–2.0)	Ref.	–

The covariates included fiscal year, age, sex, Charlson Comorbidity Index, Japan Coma Scale score at admission, location before hospitalization, admission classification, length of hospital stay before IMCU admission, main etiology of admission, and organ support therapy at IMCU admission.

IMCU, intermediate care unit; ICU, intensive care unit; CI, confidence interval; LSTs, life-sustaining therapies; IMV, invasive mechanical ventilation.

Supplementary Figure 1. Transfers between IMCUs and ICUs at the hospital level in fiscal year 2016. Among 296 hospitals with both IMCUs and ICUs in 2016, the median (interquartile range) transfers between IMCUs and ICUs, step-down transfers from the ICU to the IMCU, and step-up transfers from the IMCU to the ICU were 13.4% (5.3%–24.2%), 10.3% (2.5%–20.3%), and 2.6% (1.7%–3.5%), respectively. IMCU, intensive care unit; ICU, intermediate care unit.

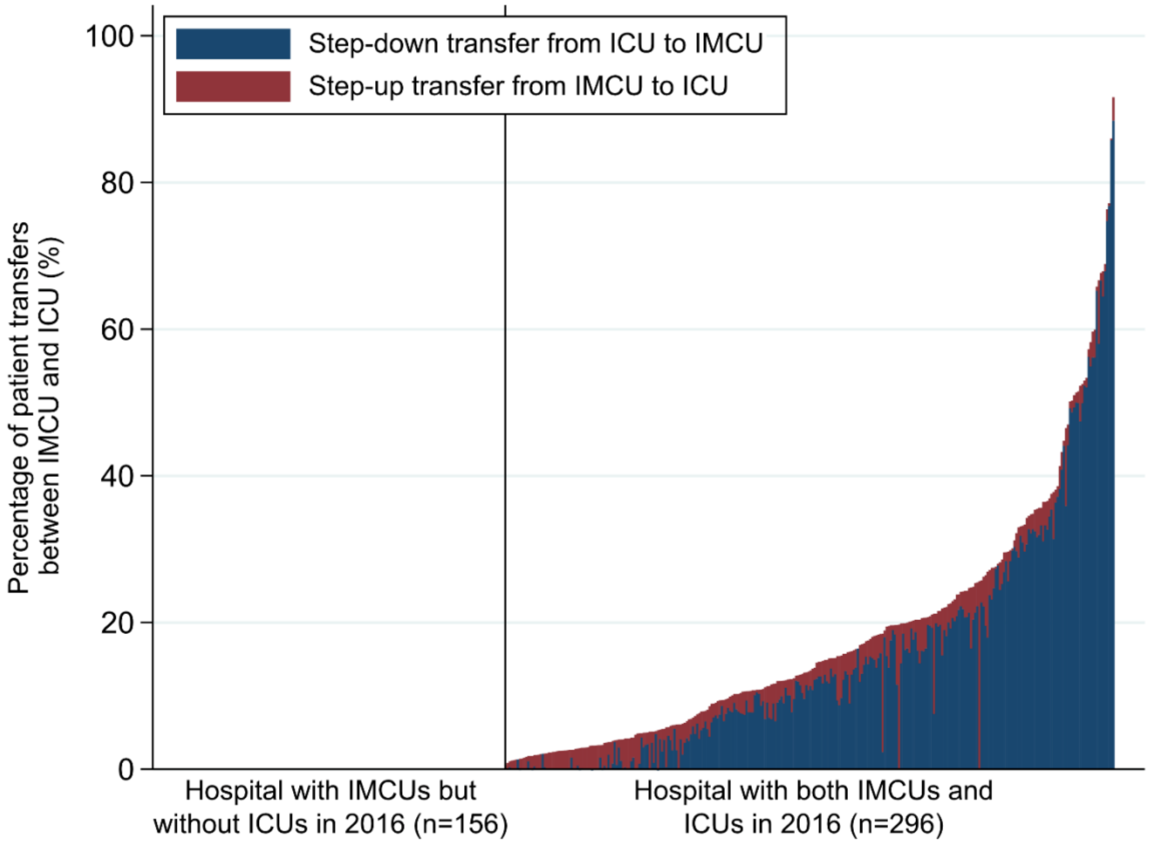

Supplementary Figure 2. Transfers between IMCUs and ICUs at the hospital level in fiscal year 2022. Among the 275 hospitals with both IMCUs and ICUs in 2022, the median (interquartile range) transfers between IMCUs and ICUs, step-down transfers from the ICU to the IMCU, and step-up transfers from the IMCU to the ICU were 10.3% (4.1%–19.2%), 7.5% (1.8%–16.5%), and 2.4% (1.6%–3.4%), respectively. IMCU, intensive care unit; ICU, intermediate care unit.

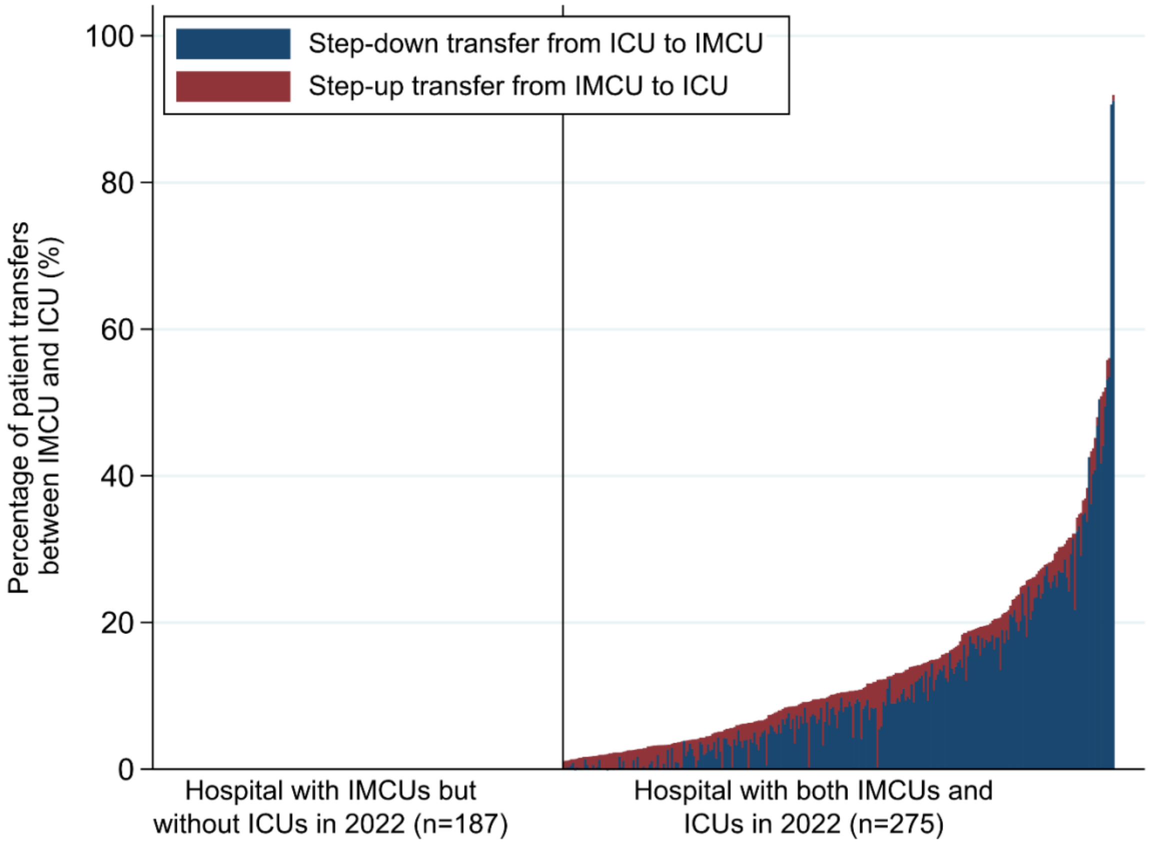

Supplement: Supplementary file 1 — Additional file1 (PDF 418 KB) [file 13054_2025_5275_MOESM1_ESM.pdf]
